# Supplementary material for: An integrated in silico-in vitro approach for identifying therapeutic targets against osteoarthritis
Source: BMC Biol. 2022 Nov 9;20:253. doi: 10.1186/s12915-022-01451-8 (PMC9648005; doi:10.1186/s12915-022-01451-8)
Supplement: Supplementary file 1 — Additional file 1: Table S1. Definitions. Definitions of technical terms pertaining to biological signaling network modeling with a semi-quantitative additive formalism. [file 12915_2022_1451_MOESM1_ESM.docx]

Table S1. Table of definitions.

| **Definitions** |
| --- |
| **Additive model**: A network of protein or gene regulation is modeled with an additive method if the evolution of variables (proteins or genes) at the next time step is defined by the sum of the upstream activating variables and the subtraction of the upstream inhibitory variables of the network. For instance, in a GRN, if a transcription factor A activates a gene P while B inhibits gene P, then the evolution of the P expression level at the next time step is defined as: $P(t+1) = A(t) - B(t)$ (eq. 1) |
| **Node or component (variable):** The nodes of a regulatory network are located at the intersections of multiple interactions (edges) in the network. They represent biological components such as proteins or genes. In mathematical models, such as the additive models, components’ evolutions are described with variables (e.g. $A$, $P$ or $B$ in (eq.1) ). |
| **Fast and Slow reactions & variables:** All reactions related to slow biological processes such as gene expression, mRNA or protein production, were referred to as slow reactions (lower priority) and those related to fast processes such as protein activation (e.g. post transcriptional modification) or degradation, were referred to as fast reactions (higher priority).  This priority order plays a role in the simulation since any biological factors could be regulated both at the protein and gene level, as in real life. Therefore, each variable was split into a fast and a slow subpart, also called sub-variables. Let’s consider that the gene P from (eq.1) produces a protein that is both activated post-transcriptionally by another protein kinase K and blocked by an inhibitory protein I. The global functional activity of the protein P is actually the multiplication of the slow by the fast subparts:  $P(t+1) = [ A(t) - B(t) ]\times[ K(t) - I(t) ]$. (eq2)  When simulating the system, the sub-variables are updated asynchronously following the priority classes in such a way that fast reactions are always updated before the slow reactions (*20*). |
| **Stable state and attractors:** The attractors of a system are the stable states (singleton attractors) and oscillatory states (cyclic attractors) towards which the system converges upon simulation. A stable state (or singleton attractor) is an ensemble of values, one for each variable of the model, that meets all the rules/constraints imposed by the equations. When variables do not evolve anymore after a certain time of simulation, the system has reached a stable state. In engineering and computer science we consider that a model has converged to a stable state when it has found a solution with an error smaller than the tolerance. In addition, two final states are considered to be the same stable state (single attractor) when the absolute difference is less than a tolerance for all variables. The nature of such a state depends on the system of equations and on which initial state was used. Given the ensemble of interconnected signaling pathways, it is likely that only a finite number of biologically distinct states can fulfil all the constraints imposed by the network structure (i.e. by the equations). So, several biologically distinct stable states may co-exist for the same system. When a system oscillates between several states without the possibility of settling down in any of the states, this is considered a cyclic attractor. |
| **Canalization & basin of attraction:** When initializing the system, it evolves over pseudo-time steps until reaching an attractor. When sampling multiple initializations (Monte Carlo analysis), it is possible to evaluate the amount of initial states reaching each attractor. This is called a canalization. The ensemble of initial states leading to an attractor constitute its basin of attraction. The size of the basin of attraction (canalization) gives a sense of the probability of reaching the attractor. |

Modelling terms and concepts relative to the current additive regulatory network model are defined in the table.
