# Supplementary material for: An integrated in silico-in vitro approach for identifying therapeutic targets against osteoarthritis
Source: BMC Biol. 2022 Nov 9;20:253. doi: 10.1186/s12915-022-01451-8 (PMC9648005; doi:10.1186/s12915-022-01451-8)
Supplement: Supplementary file 2 — Additional file 2. Supplementary computational method. Equations, mathematical framework and justification of deviation from the general rule in the equations. [file 12915_2022_1451_MOESM2_ESM.docx]

**Additional file 1. Supplementary computational method**

**Supplementary Text**

Mathematical framework and system of equations for the articular chondrocyte regulatory network:

- *Theoretical framework:*

The potential function of a component (or node) on its downstream neighbours is called the global activity. It corresponds to the multiplication of the gene activation level by the protein activation level.

Let’s consider the adjacency matrix *A* of the network whose entries *a_ij_* can be 0, 1 or -1. This matrix indicates the presence and the direction of edges in the network. If node *j* activates node *i,* then *a_ij_* is 1; if node *j* inhibits node *i*, *a_ij_* is -1. *a_ij_* equals 0 when no interaction from node *j* to node *i* is present. The matrix $L= l_{ij}$ contains a weight for each interaction in the regulatory network. Given *A* and *L*, the full system of equations contains equations for both the fast and the slow reactions regulating each variable and can be written as:

(eq.1)

$$\left\{ \begin{aligned} z_{1}^{f}\left( t+1 \right)=a_{11}^{f}l_{11}^{f} z_{1}\left( t \right)+a_{12}^{f}l_{12}^{f} z_{2}(t)\ldots+a_{1n}^{f}l_{1n}^{f}z_{n}(t) \\ \ldots\\ z_{n}^{f}\left( t+1 \right)=a_{n1}^{f}l_{n1}^{f}z_{1}\left( t \right)+a_{n2}^{f}l_{n2}^{f}z_{2}\left( t \right)\ldots+a_{nn}^{f}l_{nn}^{f} z_{n}\left( t \right) \\ \\ z_{1}^{s}\left( t+1 \right)=a_{11}^{s}l_{11}^{s} z_{1}(t) +a_{12}^{s}l_{12}^{s}z_{2}(t)\ldots+a_{1n}^{s}l_{1n}^{s}z_{n}(t) \\ \ldots\\ z_{n}^{s}\left( t+1 \right)=a_{n1}^{s}l_{n1}^{s}z_{1}\left( t \right)+a_{n2}^{s}l_{n2}^{s}z_{2}\left( t \right)\ldots+a_{nn}^{s}l_{nn}^{s} z_{n}(t) \end{aligned} \right.$$

Or

(eq.2)

$$\left\{ \begin{aligned} {\boldsymbol{z}_{\boldsymbol{i}}}^{f}\left( t+1 \right)={(A^{f}\circ L^{f}\boldsymbol{)}}_{\boldsymbol{ij}}\boldsymbol{z}_{\boldsymbol{j}}\left( t \right) \\ {\boldsymbol{z}_{\boldsymbol{i}}}^{s}\left( t+1 \right)={{(A}^{s}\circ L^{s}\boldsymbol{)}}_{\boldsymbol{ij}} \boldsymbol{z}_{\boldsymbol{j}}\left( t \right) \end{aligned} \right.$$

and the global activity of the component *i* at the time *t+1* is defined as

$$\boldsymbol{z}_{\boldsymbol{i}}\left( \boldsymbol{t+1} \right)\boldsymbol{=}\boldsymbol{z}_{\boldsymbol{i}}^{\boldsymbol{f}}\boldsymbol{(t+1)\times}\boldsymbol{z}_{\boldsymbol{i}}^{\boldsymbol{s}}\boldsymbol{(t+1)}$$

(eq.3)

where *n* is the total number of nodes in the network, **z** is a vector containing the activities for all nodes,$A^{v}={[a}_{ij}^{v}]\mathrm{and}L^{v}=\left[ l_{ij}^{v} \right]are matrices with v\in\left\{ s,f \right\} and i,j\in[1,n])$. *f* and *s* denote fast and slow variables, respectively. $z^{f}$ and $z^{s}$ and $\boldsymbol{z}$ are $n\times1$ vectors filled with the $z_{i}^{f}, z_{i}^{s} or z_{i}$ elements respectively. When two nodes are known to act in complex and in synergy, their individual terms are merged. Indeed, a term $a_{i(j,k)}l_{i(j,k)}z_{j}z_{k}$replaces $a_{ij}l_{ij}z_{j}+ a_{ik}l_{ik}z_{k}$ in the equations (**eq.1**).

The additive sum includes a weight (*l*) for each term that assumes a limited amount of saturation whenever there is a majority of stimulatory interactions. The value of that saturation depends on the interactions’ signs as defined by Kerkhofs et al. in [1]:

(eq.4)

$$l_{ij}=\left\{ \begin{aligned} 1, &if \sum_{j\in U} a_{ij}\leq1 \\ 2\frac{SC}{\sum_{j\in U} a_{ij}}, &if \sum_{j\in U} a_{ij}>1 \end{aligned} \right.$$

where *U* is the set of nodes’ indexes that are upstream node *i* and *SC* is the saturation constant. If the positive interactions do not outnumber the inhibitory interactions by more than 1, all weights are set to 1. If the number of excess positive interactions is higher than 1, we introduce a saturation factor whose value will determine how fast the node will saturate. The saturation constant was set to $\frac{2}{3}$ in that study based on [1]. The table below (**Table sup. text 1**) shows the weights for various values of $\sum_{j\in U} a_{ij}$ (the number of excess positive interactions). We refer the reader to the paper of Kerkhofs et al. [1] for more information pertaining to the implication of the saturation term and sensitivity to the saturation constant value.

| $\sum_{j\in U} a_{ij}$ (excess positive interactions) | 2 | 3 | 4 | 5 |
| --- | --- | --- | --- | --- |
| $l_{ij}$  (weight) | $\frac{2}{3}\approx0.66$ | $\frac{4}{9}\approx0.44$ | $\frac{1}{3}\approx0.33$ | $\frac{4}{15}\approx0.267$ |

**Table sup. text 1**: Weight for various values of the number of excess positive interactions and for the saturation constant set to 2/3.

Overall, while these rules cover a standard situation, incorporation of relevant biological facts should always prevail over these a priori rules. For that reason, some adaptations to the weights in the chondrocyte network were introduced to better reflect natural expression profiles in the WT situation.

Importantly, this additive model was semi-quantitative since the nodes or variables could take on a continuous activity value between 0 and 1. For a given sub-variable (i.e. fast or slow) if the sum and substraction of activator and inhibitory influences was higher than 1 (respectively lower than 0), then the component was considered as fully activated (resp. inhibited) and the value was brought back to exactly 1 (resp. 0). In mathematical terms:

(eq.5)

$$z^{v}(t)=\left\{ \begin{aligned} 0, &if z^{v}(t)\leq0 \\ 1, &if z^{v}(t)\geq1 \end{aligned} \right.$$

with $v\in\left\{ s,f \right\}$.

- *Application to the chondrocyte network:*

In the articular chondrocyte regulatory network (c.f. **Fig. 1**), the global activity of a variable $i$is defined as follow (c.f. **eq.3**) :

(eq.6)

$$\boldsymbol{z}_{\boldsymbol{i}}\left( \boldsymbol{t} \right)\boldsymbol{=}\boldsymbol{z}_{\boldsymbol{i}}^{\boldsymbol{f}}\boldsymbol{(t)\times}\boldsymbol{z}_{\boldsymbol{i}}^{\boldsymbol{s}}\boldsymbol{(t)}$$

with $i\in[1, 60]$, indicating the variable number.

The full system of reaction is provided in two parts: (a) the fast reactions, (b) the slow reactions, the variable index refers to the index provided in **Table S2**.

1. Fast reactions (protein signaling network)

$$z_{1}^{f}\left( t+1 \right)=1-z_{49}\left( t \right)-z_{48}\left( t \right)$$

$$z_{2}^{f}\left( t+1 \right)=z_{50}\left( t \right)-{0.3*z}_{4}\left( t \right)$$

$$z_{3}^{f}\left( t+1 \right)=1$$

$$z_{4}^{f}\left( t+1 \right)=z_{52}\left( t \right)-{0.5*z}_{25}\left( t \right)+ z_{52}\left( t \right)$$

$$z_{5}^{f}\left( t+1 \right)=1$$

$$z_{6}^{f}\left( t+1 \right)={\frac{2}{3}*z}_{5}\left( t \right)$$

$$z_{7}^{f}\left( t+1 \right)=1-z_{36}\left( t \right)$$

$$z_{8}^{f}\left( t+1 \right)=z_{7}\left( t \right)- z_{10}\left( t \right)$$

$$z_{9}^{f}\left( t+1 \right)=z_{38}\left( t \right)+ z_{22}\left( t \right)+ z_{32}\left( t \right)- z_{10}\left( t \right) - z_{14}\left( t \right)- z_{26}\left( t \right)\times z_{30}\left( t \right)- z_{31}\left( t \right)- z_{43}\left( t \right)$$

$$z_{10}^{f}\left( t+1 \right)=z_{14}\left( t \right)+ z_{26}\left( t \right)- z_{7}\left( t \right)- z_{19}\left( t \right)$$

$$z_{11}^{f}\left( t+1 \right)=1$$

$$z_{12}^{f}\left( t+1 \right)=z_{11}\left( t \right)$$

$$z_{13}^{f}\left( t+1 \right)=1$$

$$z_{14}^{f}\left( t+1 \right)=\left( z_{12}\left( t \right)+ z_{3}\left( t \right) \right)\times s_{2}$$

$$z_{15}^{f}\left( t+1 \right)=z_{34}\left( t \right)+ z_{19}\left( t \right)- z_{30}\left( t \right)- z_{26}\left( t \right)$$

$$z_{16}^{f}\left( t+1 \right)=1$$

$$z_{17}^{f}\left( t+1 \right)=z_{16}\left( t \right)$$

$$z_{18}^{f}\left( t+1 \right)={( z}_{17}\left( t \right)+z_{27}\left( t \right) + 0.75\times z_{42}\left( t \right))\times s_{3}- z_{19}\left( t \right)+ z_{54}\left( t \right)\times(1- z_{17}\left( t \right) )$$

$$z_{19}^{f}\left( t+1 \right)=z_{4}\left( t \right)-0.25\times z_{22}\left( t \right)$$

$$z_{20}^{f}\left( t+1 \right)=1$$

$$z_{21}^{f}\left( t+1 \right)=1$$

$$z_{22}^{f}\left( t+1 \right)=z_{41}\left( t \right)-z_{37}\left( t \right)+z_{55}\left( t \right)$$

$$z_{23}^{f}\left( t+1 \right)=1$$

$$z_{24}^{f}\left( t+1 \right)=1$$

$$z_{25}^{f}\left( t+1 \right)=1$$

$$z_{26}^{f}\left( t+1 \right)=z_{53}\left( t \right)-0.5\times\left( \left( z_{22}\left( t \right)+ z_{31}\left( t \right)+z_{25}\left( t \right) \right)\times s_{3} \right)$$

$$z_{27}^{f}\left( t+1 \right)=z_{16}\left( t \right)$$

$$z_{28}^{f}\left( t+1 \right)=\left( z_{34}\left( t \right)+ z_{34}\left( t \right)\times z_{26}\left( t \right) \right)\times s_{2}$$

$$z_{29}^{f}\left( t+1 \right)=\left( z_{27}\left( t \right)+ z_{38}\left( t \right)+z_{55}\left( t \right)- z_{58}\left( t \right) \right)\times s_{2}$$

$$z_{30}^{f}\left( t+1 \right)=z_{37}\left( t \right)-z_{35}\left( t \right)$$

$$z_{31}^{f}\left( t+1 \right)=1-0.5\times\left( z_{35}\left( t \right)+z_{18}\left( t \right) \right)$$

$$z_{32}^{f}\left( t+1 \right)=z_{34}\left( t \right)+ z_{19}\left( t \right)-z_{43}\left( t \right)$$

$$z_{33}^{f}\left( t+1 \right)=1- z_{47}\left( t \right)$$

$$z_{34}^{f}\left( t+1 \right)=\left( z_{23}\left( t \right)+ z_{33}\left( t \right)+z_{55}\left( t \right) \right)\times s_{3}$$

$$z_{35}^{f}\left( t+1 \right)=1- z_{14}\left( t \right)- z_{38}\left( t \right)$$

$$z_{36}^{f}\left( t+1 \right)=1+ z_{37}\left( t \right)- z_{2}\left( t \right)-0.5\times z_{22}\left( t \right)$$

$$z_{37}^{f}\left( t+1 \right)=z_{14}\left( t \right)$$

$$z_{38}^{f}\left( t+1 \right)=z_{39}\left( t \right)-0.5\times z_{37}\left( t \right)$$

$$z_{39}^{f}\left( t+1 \right)=\left( z_{41}\left( t \right)+ z_{42}\left( t \right)+z_{54}\left( t \right) \right)\times s_{3}$$

$$z_{40}^{f}\left( t+1 \right)=\left( z_{22}\left( t \right)+ z_{56}\left( t \right) \right)\times s_{2}$$

$$z_{41}^{f}\left( t+1 \right)=\left( z_{1}\left( t \right)+ z_{33}\left( t \right)+ z_{17}\left( t \right)+ z_{27}\left( t \right) \right)\times s_{4}$$

$$z_{42}^{f}\left( t+1 \right)=z_{3}\left( t \right)$$

$$z_{43}^{f}\left( t+1 \right)={1- z}_{32}\left( t \right)$$

$$z_{44}^{f}\left( t+1 \right)=1$$

$$z_{45}^{f}\left( t+1 \right)=\left( z_{14}\left( t \right)+ z_{22}\left( t \right) \right)\times s_{2}$$

$$z_{46}^{f}\left( t+1 \right)=1$$

$$z_{47}^{f}\left( t+1 \right)=1$$

$$z_{48}^{f}\left( t+1 \right)=1$$

$$z_{49}^{f}\left( t+1 \right)=1$$

$$z_{50}^{f}\left( t+1 \right)=z_{1}\left( t \right)- z_{49}\left( t \right)$$

$$z_{51}^{f}\left( t+1 \right)=1$$

$$z_{52}^{f}\left( t+1 \right)=z_{23}\left( t \right)- z_{33}\left( t \right)$$

$$z_{53}^{f}\left( t+1 \right)=z_{23}\left( t \right)$$

$$z_{54}^{f}\left( t+1 \right)=z_{51}\left( t \right)- z_{59}\left( t \right)$$

$$z_{55}^{f}\left( t+1 \right)=z_{54}\left( t \right)- {0.5\times z}_{34}\left( t \right)$$

$$z_{56}^{f}\left( t+1 \right)=z_{55}\left( t \right)$$

$$z_{57}^{f}\left( t+1 \right)={1-z}_{24}\left( t \right)$$

$$z_{58}^{f}\left( t+1 \right)={1-z}_{55}\left( t \right)$$

$$z_{59}^{f}\left( t+1 \right)=1$$

$$z_{60}^{f}\left( t+1 \right)=0.75 {+ z}_{56}\left( t \right)-z_{38}\left( t \right)-z_{22}\left( t \right)$$

1. Slow reactions (gene regulatory network)

$$z_{1}^{s}\left( t+1 \right)=2\times z_{6}\left( t \right)-z_{43}\left( t \right)$$

$$z_{2}^{s}\left( t+1 \right)=1$$

$$z_{3}^{s}\left( t+1 \right)=z_{14}\left( t \right)$$

$$z_{4}^{s}\left( t+1 \right)={1- z}_{45}\left( t \right)+z_{44}\left( t \right)$$

$$z_{5}^{s}\left( t+1 \right)=\left( z_{9}\left( t \right)+ z_{19}\left( t \right)+z_{29}\left( t \right)+z_{45}\left( t \right)-z_{44}\left( t \right)-z_{17}\left( t \right) \right)\times s_{2}$$

$$z_{6}^{s}\left( t+1 \right)={1- z}_{17}\left( t \right)$$

$$z_{7}^{s}\left( t+1 \right)=1$$

$$z_{8}^{s}\left( t+1 \right)=\left( 1+z_{9}\left( t \right)+ z_{7}\left( t \right) \right)\times s_{3}$$

$$z_{9}^{s}\left( t+1 \right)=\left( z_{46}\left( t \right)+ z_{8}\left( t \right)+z_{9}\left( t \right)+z_{15}\left( t \right)+z_{32}\left( t \right)-z_{21}\left( t \right)\times z_{19}\left( t \right)-z_{43}\left( t \right)+z_{6}\left( t \right)-z_{14}\left( t \right)+z_{40}\left( t \right) \right)\times s_{4}$$

$$z_{10}^{s}\left( t+1 \right)=\left( z_{14}\left( t \right)- z_{29}\left( t \right)+z_{34}\left( t \right)+z_{29}\left( t \right)+z_{10}\left( t \right)+z_{21}\left( t \right) \right)\times s_{4}$$

$$z_{11}^{s}\left( t+1 \right)=\left( z_{6}\left( t \right)\times z_{10}\left( t \right)+z_{10}\left( t \right)+z_{26}\left( t \right) \right)\times s_{3}$$

$$z_{12}^{s}\left( t+1 \right)=\left( z_{6}\left( t \right)+z_{10}\left( t \right)+z_{19}-z_{18}\left( t \right) \right)\times s_{2}$$

$$z_{13}^{s}\left( t+1 \right)=\left( z_{9}\left( t \right)+z_{4}\left( t \right)+z_{15}\left( t \right)-z_{14}\left( t \right)-z_{11}\left( t \right)+z_{46}\left( t \right)-z_{18}\left( t \right)+z_{7}\left( t \right) \right)\times s_{2}$$

$$z_{14}^{s}\left( t+1 \right)=1$$

$$z_{15}^{s}\left( t+1 \right)=\left( z_{9}\left( t \right)+z_{19}\left( t \right) \right)\times s_{2}$$

$$z_{16}^{s}\left( t+1 \right)=\left( z_{7}\left( t \right)+z_{9}\left( t \right) \right)\times s_{2}$$

$$z_{17}^{s}\left( t+1 \right)=z_{10}\left( t \right)-z_{40}\left( t \right)\times z_{22}\left( t \right)$$

$$z_{18}^{s}\left( t+1 \right)=1$$

$$z_{19}^{s}\left( t+1 \right)=1$$

$$z_{20}^{s}\left( t+1 \right)=\left( z_{10}\left( t \right)+z_{27}\left( t \right)+z_{21}\left( t \right)-z_{7}\left( t \right)+z_{60}\left( t \right) \right)\times s_{3}$$

$$z_{21}^{s}\left( t+1 \right)=\left( z_{10}\left( t \right)+z_{14}\left( t \right) \right)\times s_{2}$$

$$z_{22}^{s}\left( t+1 \right)=z_{15}\left( t \right)$$

$$z_{23}^{s}\left( t+1 \right)=z_{6}\left( t \right)-z_{40}\left( t \right)+z_{26}\left( t \right)$$

$$z_{24}^{s}\left( t+1 \right)=z_{9}\left( t \right)+z_{29}\left( t \right)+z_{46}\left( t \right) +z_{6}\left( t \right)+z_{46}\left( t \right)\times z_{9}\left( t \right)\times z_{34}\left( t \right)+z_{19}\left( t \right)+z_{40}\left( t \right)-z_{26}\left( t \right)+z_{40}\left( t \right)\times z_{28}\left( t \right)\times z_{56}\left( t \right) -{\frac{1}{5}z}_{60}\left( t \right)$$

$$z_{25}^{s}\left( t+1 \right)=\left( z_{18}\left( t \right)+z_{19}\left( t \right)+z_{26}\left( t \right)+{2\times z}_{29}\left( t \right) \right)\times s_{5}$$

$$z_{26}^{s}\left( t+1 \right)=1$$

$$z_{27}^{s}\left( t+1 \right)=z_{9}\left( t \right)-{0.5\times z}_{19}\left( t \right)$$

$$z_{29}^{s}\left( t+1 \right)=1$$

$$z_{30}^{s}\left( t+1 \right)=1$$

$$z_{31}^{s}\left( t+1 \right)=\left( z_{28}\left( t \right)+z_{6}\left( t \right)+{1.5\times z}_{14}\left( t \right)+{z_{60}\left( t \right)\times z}_{26}\left( t \right) \right)\times s_{4}$$

$$z_{32}^{s}\left( t+1 \right)=z_{15}\left( t \right)+z_{19}\left( t \right)-z_{26}\left( t \right)$$

$$z_{33}^{s}\left( t+1 \right)=\left( z_{6}\left( t \right)+z_{29}\left( t \right) \right)\times s_{2}$$

$$z_{34}^{s}\left( t+1 \right)=1-{0.4\times z}_{44}\left( t \right)$$

$$z_{35}^{s}\left( t+1 \right)=1$$

$$z_{36}^{s}\left( t+1 \right)=1$$

$$z_{37}^{s}\left( t+1 \right)=\left( 1-z_{7}\left( t \right) \right)\times s_{2}$$

$$z_{38}^{s}\left( t+1 \right)=z_{9}\left( t \right)$$

$$z_{39}^{s}\left( t+1 \right)=z_{9}\left( t \right)$$

$$z_{40}^{s}\left( t+1 \right)=1$$

$$z_{41}^{s}\left( t+1 \right)=1$$

$$z_{42}^{s}\left( t+1 \right)=\left( z_{26}\left( t \right)+z_{29}\left( t \right)+z_{18}\left( t \right) \right)\times s_{3}$$

$$z_{43}^{s}\left( t+1 \right)=\left( z_{19}\left( t \right)+z_{26}\left( t \right)+z_{43}\left( t \right)-z_{32}\left( t \right) \right)\times s_{2}$$

$$z_{44}^{s}\left( t+1 \right)=\left( z_{26}\left( t \right)+z_{29}\left( t \right)+z_{40}\left( t \right)-z_{19}\left( t \right)+z_{4}\left( t \right) \right)\times s_{3}$$

$$z_{45}^{s}\left( t+1 \right)=1$$

$$z_{46}^{s}\left( t+1 \right)=z_{29}\left( t \right)$$

$$z_{47}^{s}\left( t+1 \right)=z_{10}\left( t \right)-z_{7}\left( t \right)$$

$$z_{48}^{s}\left( t+1 \right)={0.5\times z}_{19}\left( t \right)-z_{7}\left( t \right)+z_{10}\left( t \right)-z_{29}\left( t \right)-z_{28}\left( t \right)$$

$$z_{49}^{s}\left( t+1 \right)={0.5\times z}_{19}\left( t \right)-z_{7}\left( t \right)+z_{10}\left( t \right)-z_{29}\left( t \right)-z_{28}\left( t \right)$$

$$z_{50}^{s}\left( t+1 \right)=1$$

$$z_{51}^{s}\left( t+1 \right)={z_{40}\left( t \right)\times z}_{28}\left( t \right){\times z}_{56}\left( t \right)+z_{29}\left( t \right)$$

$$z_{52}^{s}\left( t+1 \right)=\left( {0.4+z}_{8}\left( t \right) {- z}_{26}\left( t \right)+z_{9}\left( t \right)+z_{28}\left( t \right){\times z}_{56}\left( t \right) \right)\times s_{3}$$

$$z_{53}^{s}\left( t+1 \right)=\left( {0.4+z}_{26}\left( t \right) {- z}_{29}\left( t \right)+z_{10}\left( t \right) \right)\times s_{2}$$

$$z_{54}^{s}\left( t+1 \right)={1- z}_{60}\left( t \right)$$

$$z_{55}^{s}\left( t+1 \right)=1$$

$$z_{56}^{s}\left( t+1 \right)=1$$

$$z_{57}^{s}\left( t+1 \right)=\left( z_{10}\left( t \right) {+ z}_{60}\left( t \right)-z_{29}\left( t \right) \right)\times s_{3}$$

$$z_{58}^{s}\left( t+1 \right)=z_{29}\left( t \right)$$

$$z_{59}^{s}\left( t+1 \right)=\left( z_{42}\left( t \right) {+ z}_{17}\left( t \right) \right)\times s_{3}$$

$$z_{60}^{s}\left( t+1 \right)={z_{26}\left( t \right)- z}_{40}\left( t \right){\times z}_{28}\left( t \right)-z_{29}\left( t \right)$$

With $s_{p}=2 \frac{SC}{p}$, where *SC* is the saturation constant set to 2/3 and *p* is the number of excess positive interactions (see equation **eq.4**).

**Justification and deviation from general rule:** For some biological components, we lacked information regarding possible upstream regulators at the transcriptional level while information was available for the post-translational regulation, or the other way around. In other cases, post-translational modification (PTM) was simply not relevant: this is the case of ‘end nodes’ such as the ECM constitutive proteins COL-II and COL-X, which do not have any enzymatic or transcriptional activity and therefore no PTMs are relevant for regulation of downstream targets in the current system. In all those cases the sub-variable was set to 1 in the subnetwork in which no upstream regulator was describe, thereby assuming a constitutive expression or a basal protein activity level. This way, we ensure that the global activity, which is the product of the fast and slow sub-variables, is not null and that solely the effect of regulators in the subnetwork in which information is available/relevant is taken into account.

The additive rules allow that each upstream regulators can have an effect on the downstream target independently. The exceptions with multiplicative terms represent cases where multiple regulators are required concomitantly for the effect to be active on the downstream targets (similar to AND gates in Boolean models). The exceptions present in the current model are further described below. Literature support for all other equation terms (including exceptions) can be found in the Cell Collective networks (model knowledge base) [2, 3]

Justification and assumptions for exceptions in equations:

- DSH $\left( z_{2}^{f} \right):$ “$-{0.3*z}_{4}\left( t \right)$”

A weight constant was used for the inhibitor of DSH, R-SMAD ($z_{4}\left( t \right)$) since SMAD1 is thought to bind to DSH and limit its function in translating the WNT pathway [4]. Not enough information was found about other functional regulations of DSH, giving to that inhibition and strong weight, although it is not realistic that the sole presence of BMP pathway completely annihilates and blocks the WNT pathway in the model since those two pathways can be co-activated in chondrocytes, for instance during OA [4, 5]. Therefore, that inhibitory effect shouldn’t be overweighted, hence the introduction of the weight, which could be subject to sensitivity analysis.

- RUNX2 ($z_{9}^{f}$) : “$- z_{26}\left( t \right)\times z_{30}\left( t \right)”$

“The Runx-recognition-sequence context defines Smad-mediated activation versus repression. The MH1 domain of Smad3 can interact with histone deacetylases (HDACs) ($z_{30}$). SMAD3 ($z_{26}$) can recruit transcriptional activators (acetylases) or repressors like histone deacetylases [6, 7]. TGF-beta inhibited the expression of the cbfa1 and osteocalcin genes, whose expression is controlled by CBFA1 in osteoblast-like cell lines. This inhibition was mediated by SMAD3, which interacts physically with CBFA1 and represses its transcriptional activity [8, 9]. Runt-related transcription factor 2 (RUNX2) is also known as core-binding factor subunit alpha-1 (CBF-alpha-1). “HDACs are reported to interact with Runx2 and modulate Runx2 stability and transcriptional activity”, “Both HDAC4 and HDAC5 were shown to be required in TGF-β/Smad3-mediated inhibition of Runx2 activity “ [10, 11]. Therefore, the recruitment of histone deacetylase by SMAD3 to negatively regulate RUNX2 function was represented by a multiplicative term in the model.

- SMAD3 ($z_{26}^{f})$: ${"z}_{53}\left( t \right)-0.5\times\left( \left( z_{22}\left( t \right)+ z_{31}\left( t \right)+z_{25}\left( t \right) \right)\times s_{3} \right)"$

SMAD3 is the main effector transducing the TGFB1 signal through ALK5 receptors ${(z}_{53})$in chondrocyte. This pathway is well characterized and important to activate downstream cellular functions [12]. No other upstream activators for SMAD3 in chondrocyte were found and integrated in the model. However, several negative regulators of SMAD3 are also known. Nevertheless, their strengths should remain relative (not dominant inhibitions) even though more negative than positive regulators were accounted for in the model, creating an unbalance with the general rule. For the model to behave coherently, it is important that SMAD3 could be activated in the situation where TGFB1 successfully activates ALK5. Hence the saturation factor was applied to the sum of inhibitors. In addition, putting a weight in front of the upstream inhibitors allowed to moderate their effect even more, based on the stable states profiles in the random Monte Carlo analysis. This could be subject to sensitivity analysis and/or modification in future versions.

- STAT (z_18_^f^): “$z_{54}\left( t \right)\times(1- z_{17}\left( t \right) )"$

IL-1, IL-6, and tumor necrosis factor α (TNFα) play a major pathological role in chondrocytes (rheumatoid arthritis) through NF-ĸB and JAK/STAT pathways [13]

IFNgama, IL6 and IL1 activate STATS via binding to their receptor and activation of JAK [14]. Those inflammatory cytokines act on downstream pathways through the receptor of inflammatory cytokines in the model ($z_{54})$. FGF signaling, acting through FGFR3 ($z_{17}\left( t \right)$), is hypothesized to balance that effect, which was represented by an inhibitory product (1-x) in the equations. The current model overlooks many of the fine regulatory relationships controlling the JAK/STAT pathway in chondrocyte [14] and further efforts could focus on elaborating that part of the model.

- Runx2 ($z_{9}^{s}$) : “$-z_{21}\left( t \right)\times z_{19}\left( t \right)$” (Nkx3.2 * Smad complex). Nkx3/Bapx1 is a transcriptional repressor in chondrocytes. Nkx3/Bapx1 $(z_{21}\left( t \right))$ inhibits Runx2 expression [15]. BMP signalling, of which the downstream effector is the Smad complex ($z_{19}\left( t \right)$) in the model, is required for Nkx3.2 to serve as a transcriptional repressor of chondrogenesis [16, 17].
- PTHrP ($z_{11}^{s}$): ${“z}_{6}\left( t \right)\times z_{10}\left( t \right)”$

PTHrP normally functions in a feedback loop with Indian hedgehog (IHH), in which a reduction in one signaling partner induces a compensatory increase in the other. IHH signaling promotes PTHrP production, which in turn inhibits IHH signaling [18, 19]. In the growth plate, this ensure the spatial organization of proliferating and hypertrophic chondrocytes since IHH promotes PTHrP signalling in SOX9 positive, proliferative, while in articular cartilage this might be key in the maintenance of healthy phenotype or transition towards hypertrophic changes. “Sox9 family members functionally collaborated with Ihh/Gli2 signaling to regulate PTHrP expression and chondrocyte differentiation“ [19, 20]

- FGFR3 (z_17_^s^): “$-z_{40}\left( t \right)\times z_{22}\left( t \right)”.$

FGFR3 is negatively regulated by FGF-2 at the transcriptional level through the FGFR1-ERK (extracellular signal-regulated kinase) signaling pathway in human articular chondrocytes [21, 22]. ELK1 ($z_{40})$ transduces ERK1/2 ($z_{22})$ signal into the nucleus (transcriptional effector in the model) [23]. However, simply adding an inhibitory link from ELK1 to FGR3 in the model would not be accurate to translate the intended regulation since ELK1 is located downstream of several MAPKs, hence a ERK1/2 dependent inhibition of FGFR3 by ELK1 was introduce with a multiplicative term to account for that.

- MMP13 $(z_{24}^{s})$: “  $+z_{40}\left( t \right)\times z_{28}\left( t \right)\times z_{56}\left( t \right)$ ” and “ ${+ z}_{46}\left( t \right)\times z_{9}\left( t \right)\times z_{34}\left( t \right)$”

“JNK ${(z}_{56}\left( t \right))$ pathway, which mediates phosphorylation of AP-1/Jun family members, as well as Elk-1 $z_{40}\left( t \right)$ and ATF-2 ($z_{56}\left( t \right))$, is required for cytokine induction of MMP-1 and MMP-13” [24]. ELK1 and ATF2 may also transduce the signal of other MAPKs in chondrocytes and in the model) [25], therefore a multiplicative term was introduced to ensure the aforementioned observations was well represented (involvement of JNK pathway specifically)

The exact role of p38 ($z_{34}\left( t \right))$ in chondrocyte is not straightforward and remains poorly understood. Seemingly, contradictory results exist in the literature. In physiological conditions, p38 may have a protective effect against cartilage degradation through downregulation of collagen X [12, 26]. On the other hand, some results show that p38 is related to hypertrophic differentiation and induced MMP13 expression is dependent upon p38 activation in OA [26]. However, “p38 signaling alone may not largely affect MMP13 expression in chondrocytes” [12]. Finally p38 is more expressed and active in OA than normal cartilage [23]. To account for the fact that p38 can promote MMP13 in OA chondrocytes only, that its activity is enhanced in OA and that it requires co-factors, the conditional presence of RUNX2 ($z_{9}\left( t \right))$ and HIF2a ($z_{46}\left( t \right))$ was implemented via a multiplicative factor (this way, p38 increases MMP13 expression only if Runx2 and Hif2a are active). Indeed Runx2 and Hif2a are hallmarks of chondrocyte hypertrophy and OA [27].

- CCND1 (z_31_^s^): “${z_{60}\left( t \right)\times z}_{26}\left( t \right)"$.

FOXO would promote Cyclin D expression (FOXO KEGG pathway).

Together with ATF2, Smad3 activates TGF-beta regulated genes, including CCND1 [28]. FOXO1 ($z_{60}\left( t \right))$ was reported to regulate chondrogenic differentiation and cell cycle in collaboration with TGFB1 signaling [29, 30], which was translated by a multiplicative factor with SMAD3($z_{26}\left( t \right))$, which we assumed to be the involved co-factor since it is the downstream effector of TGFB1 in the model.

- P38 ($z_{34}^{s}):$ “$1-{0.4\times z}_{44}\left( t \right)$”

Little information was found about the transcriptional regulators of p38 in literature. Many regulatory relationships have probably been overlooked for that factor in the current model. With the general rule a value of 1 is applied in the subnetwork if no regulators are described. However, the data driven inference predicted d-EF1 as a potential transcriptional inhibitor of p38 (see Fig. 2 in the main paper). Nevertheless, it is unlikely that the sole presence of d-EF1 would suffice to annihilate the whole p38 pathway in chondrocyte. So a modulatory weight was introduced to account for the inhibitory effect of d-EF1 while not overweighting its influence. The weight was chosen arbitrarily as the general rule handle excess of positive interactions to control the saturation of a variable. This weight could be subjected to a sensitivity analysis.

- Proinflammatory cytokines ($z_{51}^{s}$) : $“{z_{40}\left( t \right)\times z}_{28}\left( t \right){\times z}_{56}\left( t \right)”$

MAPK pathways converge to upregulates expression of pro-inflammatory cytokines, such as TNFα and IL-1, during osteoarthritis [23]. Each parallel cascade has a different downstream effector, represented by ELK1 ($z_{40}\left( t \right))$, ATF2 ($z_{28}\left( t \right))$ (or CREB2) and c-JUN (not represented in the model but directly downstream of JNK $\left( z_{56}\left( t \right) \right)$ [23]. A multiplicative term was introduced to require MAPK pathways to converge for cytokines to be upregulated.

- ALKs receptors (ALK5 ($z_{53}^{s}$) and ALK1 ($z_{52}^{s}$)): constant and special terms “$z_{28}\left( t \right){\times z}_{56}\left( t \right)$”

Both receptors of TGFB are expressed in cartilage in health and disease, although the balance between the two receptors is known to changes during disease. ALK5 becomes less expressed while ALK1 becomes more abundant in OA chondrocytes [31, 32]. The ratio changes but both, should remain expressed to some basal level. However, all transcriptional activators for ALK1 that were found and included in the model were factors specifically activated during hypertrophy or inflammation while transcriptional activators for ALK5 were more active in healthy chondrocytes. This would make it virtually impossible for ALK1 and ALK5 to have a non-zero basal expression (and possible minimal level of activation) in the healthy and hypertrophic state, respectively. To avoid this bias due to the lack of information, we introduced a constitutive expression (default arbitrary constant), which could the focus of a sensitivity analysis. This constitutive expression was accounted for in the assignment of the saturation factor. Moreover, the data driven inference (Fig.2 in main text) predicted a transcriptional regulation of ALK1 by ATF2 ($z_{28}\left( t \right))$. In addition, the Gene Card database confirmed the existence of transcriptional binding domains for ATF2 and JNK-cJUN ($z_{56}\left( t \right))$ [33] in the ALK1 promoter region. ATF2 is also a known transcriptional effector downstream of the MAPK/JNK pathways. In the model, we have represented the convergence of the various MAPK/JNK pathways on common downstream effectors and targets through a multiplicative term [23]. For consistency, we hypothesized that ATF2 and JNK-cJUN work in coordination to regulate ALK1 too, so the same multiplicative term was applied.

- FOXO ($z_{60}^{s}$): “ ${- z}_{40}\left( t \right){\times z}_{28}\left( t \right)$”

Interleukin-1β or tumor necrosis factor also caused rapid downregulation of FOXO1 via JNK [34] of which ELK1 ($z_{40}\left( t \right)$) and ATF2 ($z_{28}\left( t \right)$) are downstream effectors in the model [23]. MAPK pathways are involved in FOXOs downregulation in a JNK dependent way [34]. No information was found about the exact downstream transcription factor involved in that regulation but MAPK-mediated inflammatory signal are known to converge through several parallel paths (MAPK/JNK – ELK1/ATF2/cJUN) [23], so the inhibitory effect was attributed to a joint effect (multiplicative term) of the two effectors of JNK and MAPKs in the model: ELK1 and ATF2.

All other interactions described in the model follow the general rule and are supported by literature data in the cell collective networks.

**References:**

1. Kerkhofs J, Geris L. A Semiquantitative Framework for Gene Regulatory Networks: Increasing the Time and Quantitative Resolution of Boolean Networks. PLoS One. 2015;10:e0130033.

2. Lesage R, Geris L. PPI network- Interactive Modeling of Biological Networks | Cell Collective. Cell Collective. 2021. https://doi.org/https://research.cellcollective.org/#a5b66073-6769-4c88-bf6b-37ca1aa8f766.

3. Lesage R, Geris L. GRN - Interactive Modeling of Biological Networks | Cell Collective. Cell Collective. 2021. https://doi.org/https://research.cellcollective.org/#474de240-8752-4c3b-aa63-23640e50bf7a.

4. Wang Y, Fan X, Xing L, Tian F. Wnt signaling: A promising target for osteoarthritis therapy. Cell Communication and Signaling. 2019;17:1–14.

5. Leijten JCH, Emons J, Sticht C, Van Gool S, Decker E, Uitterlinden A, et al. Gremlin 1, frizzled-related protein, and dkk-1 are key regulators of human articular cartilage homeostasis. Arthritis and Rheumatism. 2012;64:3302–12.

6. Derynck R, Zhang YE. Smad-dependent and Smad-independent pathways in TGF-β family signalling. Nature. 2003;425:577–84.

7. Liberati NT, Moniwa M, Borton AJ, Davie JR, Wang XF. An Essential Role for Mad Homology Domain 1 in the Association of Smad3 with Histone Deacetylase Activity*. Journal of Biological Chemistry. 2001;276:22595–603.

8. Wu M, Chen G, Li YP. TGF-β and BMP signaling in osteoblast, skeletal development, and bone formation, homeostasis and disease. Bone Research. 2016;4:1–21.

9. Chen CG, Thuillier D, Chin EN, Alliston T. Chondrocyte-intrinsic Smad3 represses Runx2-inducible matrix metalloproteinase 13 expression to maintain articular cartilage and prevent osteoarthritis. Arthritis and Rheumatism. 2012;64:3278–89.

10. Kang JS, Alliston T, Delston R, Derynck R. Repression of Runx2 function by TGF-β through recruitment of class II histone deacetylases by Smad3. EMBO Journal. 2005;24:2543–55.

11. Jonason JH, Xiao G, Zhang M, Xing L, Chen D. Post-translational regulation of Runx2 in bone and cartilage. Journal of Dental Research. 2009;88:693–703.

12. Li TF, Gao L, Sheu TJ, Sampson ER, Flick LM, Konttinen YT, et al. Aberrant hypertrophy in Smad3-deficient murine chondrocytes is rescued by restoring transforming growth factor β-activated kinase 1/ activating transcription factor 2 signaling: A potential clinical implication for osteoarthritis. Arthritis and Rheumatism. 2010;62:2359–69.

13. Nishimura R, Hata K, Takahata Y, Murakami T, Nakamura E, Ohkawa M, et al. Role of signal transduction pathways and transcription factors in cartilage and joint diseases. International Journal of Molecular Sciences. 2020;21:1340.

14. Aaronson DS, Horvath CM. A road map for those who don’t know JAK-STAT. Science. 2002;296:1653–5.

15. Provot S, Kempf H, Murtaugh LC, Chung U Il, Kim DW, Chyung J, et al. Nkx3.2/Bapx1 acts as a negative regulator of chondrocyte maturation. Development. 2006;133:651–62.

16. Rainbow RS, Kwon H, Zeng L. The role of Nkx3.2 in chondrogenesis. Frontiers in Biology. 2014;9:376–81.

17. Zeng L, Kempf H, Murtaugh LC, Sato ME, Lassar AB. Shh establishes an Nkx3.2/Sox9 autoregulatory loop that is maintained by BMP signals to induce somitic chondrogenesis. Genes and Development. 2002;16:1990–2005.

18. MacIca C, Liang G, Nasiri A, Broadus AE. Genetic evidence of the regulatory role of parathyroid hormone-related protein in articular chondrocyte maintenance in an experimental mouse model. Arthritis and Rheumatism. 2011;63:3333–43.

19. Chen X, Macica CM, Nasiri A, Broadus AE. Regulation of articular chondrocyte proliferation and differentiation by Indian hedgehog and parathyroid hormone-related protein in mice. Arthritis and Rheumatism. 2008;58:3788–97.

20. Amano K, Hata K, Sugita A, Takigawa Y, Ono K, Wakabayashi M, et al. Sox9 family members negatively regulate maturation and calcification of chondrocytes through up-regulation of parathyroid hormone-related protein. Molecular Biology of the Cell. 2009;20:4541–51.

21. Vincent TL. Fibroblast growth factor 2: Good or bad guy in the joint? Arthritis Research and Therapy. 2011;13:127.

22. Yan D, Chen D, Cool SM, van Wijnen AJ, Mikecz K, Murphy G, et al. Fibroblast growth factor receptor 1 is principally responsible for fibroblast growth factor 2-induced catabolic activities in human articular chondrocytes. Arthritis Research and Therapy. 2011;13:R130.

23. Loeser RF, Erickson EA, Long DL. Mitogen-activated protein kinases as therapeutic targets in osteoarthritis. Current Opinion in Rheumatology. 2008;20:581–6.

24. Goldring MB, Otero M, Tsuchimochi K, Ijiri K, Li Y. Defining the roles of inflammatory and anabolic cytokines in cartilage metabolism. In: Annals of the Rheumatic Diseases. NIH Public Access; 2008. p. iii75.

25. Kirsch K, Zeke A, Tőke O, Sok P, Sethi A, Sebő A, et al. Co-regulation of the transcription controlling ATF2 phosphoswitch by JNK and p38. Nature Communications. 2020;11:1–15.

26. Stanton LA, Sabari S, Sampaio A V., Underhill TM, Beier F. p38 MAP kinase signalling is required for hypertrophic chondrocyte differentiation. Biochemical Journal. 2004;378:53–62.

27. Nishimura R, Hata K, Nakamura E, Murakami T, Takahata Y. Transcriptional network systems in cartilage development and disease. Histochemistry and Cell Biology. 2018;149:353–63.

28. Ionescu AM, Schwarz EM, Zuscik MJ, Drissi H, Puzas JE, Rosier RN, et al. ATF-2 cooperates with Smad3 to mediate TGF-β effects on chondrocyte maturation. Experimental Cell Research. 2003;288:198–207.

29. Kurakazu I, Akasaki Y, Hayashida M, Tsushima H, Goto N, Sueishi T, et al. FOXO1 transcription factor regulates chondrogenic differentiation through transforming growth factor β1 signaling. Journal of Biological Chemistry. 2019;294:17555–69.

30. Matsuzaki T, Alvarez-Garcia O, Mokuda S, Nagira K, Olmer M, Gamini R, et al. FoxO transcription factors modulate autophagy and proteoglycan 4 in cartilage homeostasis and osteoarthritis. Science Translational Medicine. 2018;10:eaan0746.

31. Thielen NGM, Van der Kraan PM, Van Caam APM. TGFβ/BMP signaling pathway in cartilage homeostasis. Cells. 2019;8.

32. Van Der Kraan PM. The changing role of TGFβ in healthy, ageing and osteoarthritic joints. Nature Reviews Rheumatology. 2017;13:155–63.

33. GeneCards - ALK1. https://www.genecards.org/cgi-bin/carddisp.pl?gene=ACVRL1&keywords=ALK1. Accessed 20 Jun 2022.

34. Grabiec AM, Angiolilli C, Hartkamp LM, Van Baarsen LGM, Tak PP, Reedquist KA. JNK-dependent downregulation of FoxO1 is required to promote the survival of fibroblast-like synoviocytes in rheumatoid arthritis. Annals of the Rheumatic Diseases. 2015;74:1763–71.
