# Supplementary material for: An integrated in silico-in vitro approach for identifying therapeutic targets against osteoarthritis
Source: BMC Biol. 2022 Nov 9;20:253. doi: 10.1186/s12915-022-01451-8 (PMC9648005; doi:10.1186/s12915-022-01451-8)
Supplement: Supplementary file 3 — Additional file 3: Table S2. List of variables and mouse genes correspondence. All mathematical variables and the corresponding node in the network have names written in upper cases and do not reflect the official human or mouse nomenclatures. To relate those variables to actual genes more easily, we provide this table of correspondence. A related mouse gene name and NCBI ID is indicated for each variable. Nevertheless, this is not exhaustive since some variables represent a group of factors or a family of ligands rather than a single factor. [file 12915_2022_1451_MOESM3_ESM.docx]

Table S2. Table of correspondence between the model’s variables and corresponding mouse genes.

| **Variable index** | **Variable name** | **Example representative mouse gene** | |
| --- | --- | --- | --- |
|  |  | **Name** | **NCBI ID** |
| 1 | WNT | *Wnt3a* | 22416 |
| 2 | DSH | *Dvl1* | 20423 |
| 3 | IGF-I | *Igf1* | 16000 |
| 4 | R-SMAD | *Smad5* | 17129 |
| 5 | IHH | *Ihh* | 16147 |
| 6 | GLI2 | *Gli2* | 14633 |
| 7 | β-Catenin | *Ctnnb1* | 12387 |
| 8 | LEF/TCF | *Tcf7* | 21414 |
| 9 | RUNX2 | *Runx2 or Cbfa1* | 12393 |
| 10 | SOX9 | *Sox9* | 20682 |
| 11 | PTHrP | *Pthlh* | 19227 |
| 12 | PPR | *Pth1r* | 19228 |
| 13 | COL-X | *Col10a1* | 12813 |
| 14 | PKA | *Prkaca* | 18747 |
| 15 | MEF2C | *Mef2c* | 17260 |
| 16 | FGF | *Fgf2* | 14173 |
| 17 | FGFR3 | *Fgfr3* | 14184 |
| 18 | STAT1 | *Stat1* | 20846 |
| 19 | Smadcomplex | *Smad4 & R-Smads* | 17128 |
| 20 | COL II | *Col2a1* | 12824 |
| 21 | NKX3.2 | *Nkx3-2 or Bapx1* | 12020 |
| 22 | ERK1/2 | *Mapk3* | 26417 |
| 23 | TGFβ | *Tgfb1* | 21803 |
| 24 | MMP13 | *Mmp13* | 17386 |
| 25 | SMAD7 | *Smad7* | 17131 |
| 26 | SMAD3 | *Smad3* | 17127 |
| 27 | FGFR1 | *Fgfr1* | 14182 |
| 28 | ATF2 | *Atf2* | 11909 |
| 29 | NFκB | *Nfkb1* | 18033 |
| 30 | HDAC4 | *Hdac4* | 208727 |
| 31 | CCND1 | *Ccnd1* | 12443 |
| 32 | DLX5 | *Dlx5* | 13395 |
| 33 | BMP | *Bmp2* | 12156 |
| 34 | P38 | *Mapk14* | 26416 |
| 35 | GSK3β | *Gsk3b* | 56637 |
| 36 | DC | *Apc* | 11789 |
| 37 | PP2A | *Ppp2ca* | 19052 |
| 38 | AKT | *Akt1* | 11651 |
| 39 | PI3K | *Pi3kr1* | 18708 |
| 40 | ETS1 | *Ets1* | 23871 |
| 41 | RAS | *Kras* | 16653 |
| 42 | IGF-IR | *Igf1r* | 16001 |
| 43 | MSX2 | *Msx2* | 17702 |
| 44 | δEF-1 | *Zeb1* | 21417 |
| 45 | ATF4 | *Atf4 or Creb* | 11911 |
| 46 | HIF-2α | *Epas1* | 13819 |
| 47 | GREM1 | *Grem1* | 23892 |
| 48 | DKK1 | *Dkk1* | 13380 |
| 49 | FRZB | *Frzb* | 20378 |
| 50 | Frizzled-LRP5/7 | *Lrp5* | 16973 |
| 51 | Cytokines | *Il1b* | 16176 |
| 52 | ALK1 | *Acvrl1* | 11482 |
| 53 | ALK5 | *Tgrb1* | 21812 |
| 54 | R-Infl (Receptor inflammation) | *Tlr1* | 21897 |
| 55 | TAK1 | *Map3k7* | 26409 |
| 56 | JNK | *Mapk8* | 26419 |
| 57 | Proteoglycans | *Acan* | 11595 |
| 58 | IkB-a | *Nfkbi* | 18035 |
| 59 | SOCS | *Socs1* | 12703 |
| 60 | FOXO1 | *Foxo1* | 56458 |

All mathematical variables and the corresponding nodes in the network have names written in upper cases and do not reflect the official human or mouse nomenclature. To relate those variables to actual genes more easily, we provide this table of correspondence. A related mouse gene name and NCBI ID is indicated for each variable. Nevertheless, it is not exhaustive since some variables represent a group of factors or a family of ligands rather than a single factor.
