# Supplementary material for: An integrated in silico-in vitro approach for identifying therapeutic targets against osteoarthritis
Source: BMC Biol. 2022 Nov 9;20:253. doi: 10.1186/s12915-022-01451-8 (PMC9648005; doi:10.1186/s12915-022-01451-8)
Supplement: Supplementary file 5 — Additional file 5: Fig. S1. Individual dataset clustering and heatmap. The microarray sub-datasets, used in the merged dataset, were also investigated individually. They were pre-processed (i.e. processing steps before merging and correcting for batch effect) in the same way as the merged dataset but the unsupervised clustering analysis was done on each sub-dataset separately in order to compare the biological or OA related information content before and after the data merging. The heatmaps show the expression profile of the same list of genes of interest than for the merged dataset (see Additional file 4, Data S1.). These expression datasets were submitted to unsupervised clustering with the Euclidean distance method and the Complete aggregation method in R thanks to the heatmap3 function from the Github repository https://github.com/obigriffith/biostar-tutorials/tree/master/Heatmaps . The headers indicate the GEO accession numbers of the 6 original datasets. Samples labeled as ‘WT’, for wild type, are in green, samples labeled as ‘OA’, for osteoarthritis, in red. The pre-labelling is the same as for the merged dataset (see Additional file 4, Data S1). When applicable, a grey scale indicate the time points (w stands for weeks, in the legend). For some datasets (e.g. GSE26475, GSE33656, GSE53857) the OA and the WT samples are well separated in different clusters, while for other the separation is not so clear. For instance, in GSE45793 the variance due the time point 6weeks is greater than the OA induced variance, while for weeks 1 and 2 OA and WT samples are well separated due to the OA condition. [file 12915_2022_1451_MOESM5_ESM.docx]

Fig. S1. Heatmap with unsupervised clustering on individual dataset.

The microarray sub-datasets, used in the merged dataset, were also investigated individually. They were pre-processed (i.e. processing steps before merging and correcting for batch effect) in the same way as the merged dataset but the unsupervised clustering analysis was done on each sub-dataset separately in order to compare the biological or OA related information content before and after the data merging. The heatmaps show the expression profile of the same list of genes of interest than for the merged dataset (see **Additional file 4,** **Data S1**.). These expression datasets were submitted to unsupervised clustering with the Euclidean distance method and the Complete aggregation method in R thanks to the heatmap3 function from the Github repository <https://github.com/obigriffith/biostar-tutorials/tree/master/Heatmaps>. The headers indicate the GEO accession numbers of the 6 original datasets. Samples labeled as ‘WT’, for wild type, are in green, samples labeled as ‘OA’, for osteoarthritis, in red. The pre-labelling is the same as for the merged dataset (see **Additional file 4,** **Data S1**). When applicable, a grey scale indicate the time points (w stands for weeks, in the legend). For some datasets (e.g. GSE26475, GSE33656, GSE53857) the OA and the WT samples are well separated in different clusters, while for other the separation is not so clear. For instance, in GSE45793 the variance due the time point 6weeks is greater than the OA induced variance, while for weeks 1 and 2 OA and WT samples are well separated due to the OA condition.
