# Supplementary material for: An integrated in silico-in vitro approach for identifying therapeutic targets against osteoarthritis
Source: BMC Biol. 2022 Nov 9;20:253. doi: 10.1186/s12915-022-01451-8 (PMC9648005; doi:10.1186/s12915-022-01451-8)
Supplement: Supplementary file 7 — Additional file 7: Table S3. Validation of transcriptional data-inferred regulatory interactions. Transcriptional interactions were inferred from the merged OA dataset. Inference was run with three algorithms and only interactions that were present in the results of the three algorithms (GENIE3, ARACNE, TIGRESS) were considered as additions to the model. An interaction was considered present for one algorithm if it scored higher than a threshold defined as the difference between the mean and standard deviation of all scores (Additional file 6, Data S2 ). Inferred interactions were validated whenever possible by looking for binding sites of the source transcription factor in the enhancer region of the target gene with GeneCards. The complete list of GeneHancer IDs provided by GeneCards are reported in the last column. ‘NA’ indicate that no binding site was found. The exact gene names and gene IDs that were queried in GeneCards are reported in the first columns. [file 12915_2022_1451_MOESM7_ESM.docx]

Table S3. Validation of transcriptional data-inferred regulatory interactions.

| **Source** | | **Target** | | **GeneCards Validation -**  **Genehancer identifier** | **Reference** |
| --- | --- | --- | --- | --- | --- |
| **Name** | **NCBI ID** | **Name** | **NCBI ID** |  |  |
| Atf2 | 11909 | Alk1 (Acvrl1) | 11482 | GH12J051904, GH12J051969, GH12J051922, GH12J052180 | https://www.genecards.org/cgi-bin/carddisp.pl?gene=ACVRL1&keywords=ALK1 |
| Atf4 | 11911 | R-smad (Smad5) | 17129 | GH05J135132 | https://www.genecards.org/cgi-bin/carddisp.pl?gene=SMAD5&keywords=Smad5 |
| Stat1 | 20846 | Col10a1 | 12813 | GH06J11609 | https://www.genecards.org/cgi-bin/carddisp.pl?gene=COL10A1&keywords=COL10a1 |
| Zeb1 | 21417 | p38 (Mapk14) | 26416 | GH06J036116 | https://www.genecards.org/cgi-bin/carddisp.pl?gene=MAPK14&keywords=p38 |
| Atf2 | 11909 | Alk5 (Tgfbr1) | 21812 | GH09J099118, GH09J099102 | https://www.genecards.org/cgi-bin/carddisp.pl?gene=TGFBR1&keywords=ALK5 |
| Ctnnb1 | 12387 | Col10a1 | 12813 | NA |  |
| Ctnnb1 | 12387 | Col2a1 | 12824 | NA |  |
| Ctnnb1 | 12387 | Ppp2ca | 19052 | NA |  |
| Mef2c | 17260 | erk1/2 (Mapk1) | 26413 | NA |  |
| Zeb1 | 21417 | R-smad (Smad5) | 17129 | GH05J136051, GH05J135190, GH05J136619, GH05J136729 | https://www.genecards.org/cgi-bin/carddisp.pl?gene=SMAD5&keywords=smad5 |
| R-smad (Smad5) | 17129 | Zeb1 | 21417 | GH10J031316, GH10J031752, GH10J031132, GH10J031925, GH10J031696 | https://www.genecards.org/cgi-bin/carddisp.pl?gene=ZEB1&keywords=zeb1 |

Transcriptional interactions were inferred from the merged OA dataset. Inference was run with three algorithms and only interactions that were present in the results of the three algorithms (GENIE3, ARACNE, TIGRESS) were considered as additions to the model. An interaction was considered present for one algorithm if it scored higher than a threshold defined as the difference between the mean and standard deviation of all scores (**Additional file 6, Data S2** ). Inferred interactions were validated whenever possible by looking for binding sites of the source transcription factor in the enhancer region of the target gene with GeneCards. The complete list of GeneHancer IDs provided by GeneCards are reported in the last column. ‘NA’ indicate that no binding site was found. The exact gene names and gene IDs that were queried in GeneCards are reported in the first columns.
