# Supplementary material for: An integrated in silico-in vitro approach for identifying therapeutic targets against osteoarthritis
Source: BMC Biol. 2022 Nov 9;20:253. doi: 10.1186/s12915-022-01451-8 (PMC9648005; doi:10.1186/s12915-022-01451-8)
Supplement: Supplementary file 10 — Additional file 10: Fig. S2. Effect of ALKs ratio in the influence of inflammation and TGFβ signaling on chondrocyte hypertrophy. Percentage of perturbations remaining in the healthy state (right) or transitioning towards the hypertrophic one (left) during inflammatory pathway activation with TGF-B treatment while changing the ratio between ALK1 and ALK5. The inflammatory and TGF-B profiles that were imposed are the same as in Fig. 4A except that the value imposed for ALK1 and ALK5 are varying between 0 and 1 with a 0.1 increment. ALK1=ALK5 on the red diagonals and ALK1>ALK5 in the upper right corner. Roughly, the rescue of the healthy state by TGF-B is lost when ALK1 is greater than ALK5. If ALK1 is high enough and that the difference between ALK1 and ALK5 is not greater than 10-20% then the protective effect of TGF-B is also disturbed for ALK1<ALK5. [file 12915_2022_1451_MOESM10_ESM.docx]

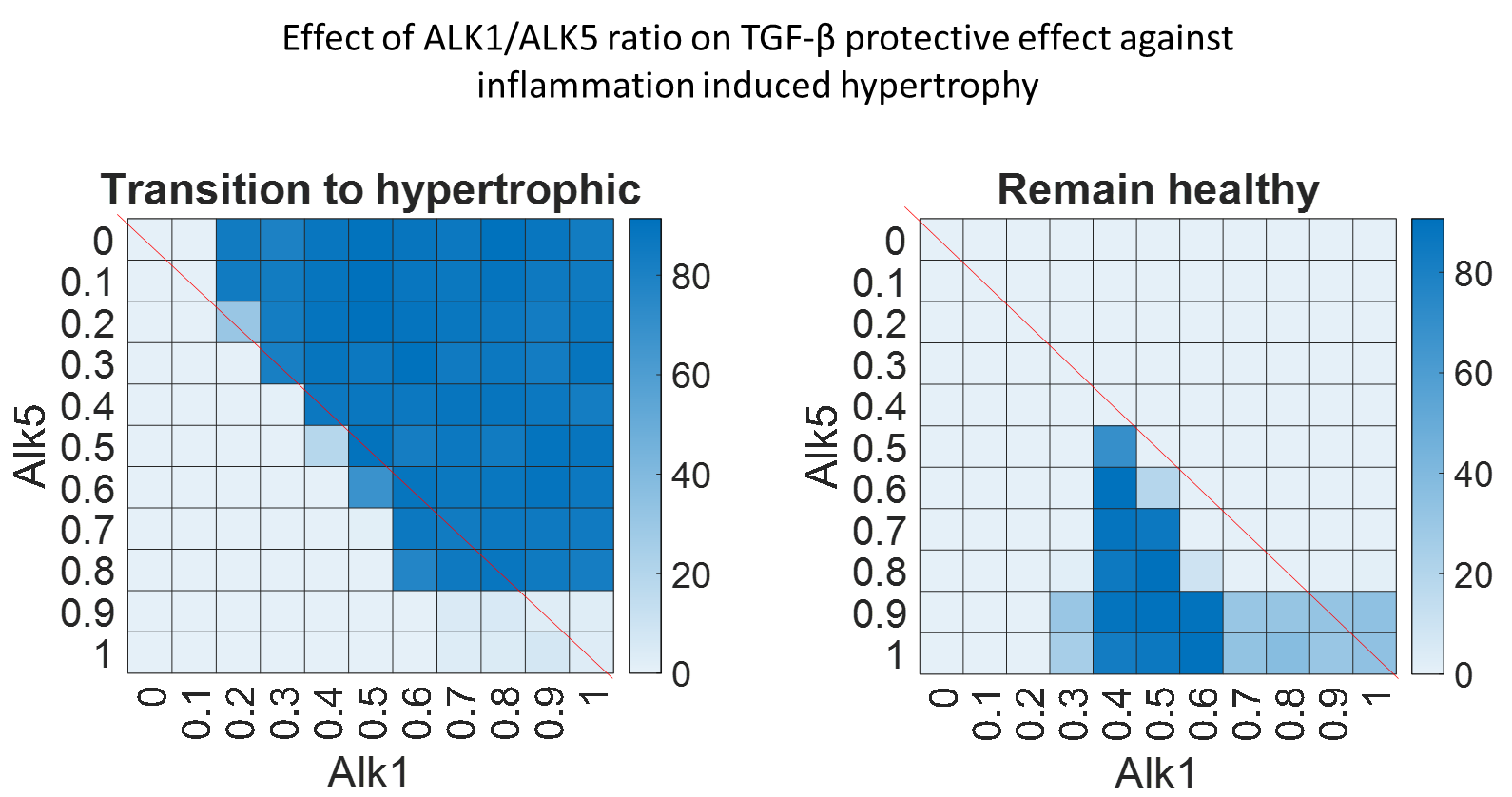
Fig. Fig S2. Role of the ratio between receptors ALK1 and ALK5 in the effect of inflammation and TGFβ signaling on chondrocyte hypertrophy

Percentage of perturbations remaining in the healthy state (right) or transitioning towards the hypertrophic one (left) during inflammatory pathway activation with TGF-B treatment while changing the ratio between ALK1 and ALK5. The inflammatory and TGF-B profiles that were imposed are the same as in Fig.4A except that the value imposed for ALK1 and ALK5 are varying between 0 and 1 with a 0.1 increment. ALK1=ALK5 on the red diagonals and ALK1>ALK5 in the upper right corner. Roughly, the rescue of the healthy state by TGF-B is lost when ALK1 is greater than ALK5. If ALK1 is high enough and that the difference between ALK1 and ALK5 is not greater than 10-20% then the protective effect of TGF-B is also disturbed for ALK1<ALK5
