# Supplementary material for: An integrated in silico-in vitro approach for identifying therapeutic targets against osteoarthritis
Source: BMC Biol. 2022 Nov 9;20:253. doi: 10.1186/s12915-022-01451-8 (PMC9648005; doi:10.1186/s12915-022-01451-8)
Supplement: Supplementary file 11 — Additional file 11: Fig. S3. Screenshot of the user-friendly interface for the virtual chondrocytes App. The standalone Matlab-based applications can be launched and used without Matlab license, provided that the compiler Matlab Runtime is installed (https://nl.mathworks.com/products/compiler/matlab-runtime.html). The virtual chondrocyte initial state can be set as healthy or hypertrophic, allowing the user to test any scenarios. All the 60 components may be perturbed alone or in any sort of combination by forcing the variables to take a value in the interval [0:1], with a step of 0.1. The most left column indicate the value of the variable in the selected initial state, for information. Obviously, applying a perturbation that is equal to the initial value of the variable will not affect the system. Once the setting are done, the user can apply the experimental condition by pushing the button ‘Test condition’ and the percentage of transitions towards each of the possible basal stable states (i.e. ‘None’, ‘Healthy’ and ‘Hypertrophic’) is computed. If the ‘Compute statistics’ box is ticked, then the experiment is repeated 3 times and the average and standard deviations are displayed (variation occurs due to the stochastic nature of the model). The results may be exported and saved in an excel file via the ‘Save’ button. The application can be installed with the executable file on the GitHub repository, [https://github.com/Rapha-L/Insilico_chondro.git]. No Matlab license is required, however, the operating system should be able to support the Matlab software. For Linux users, a Windows virtual machine may be used. [file 12915_2022_1451_MOESM11_ESM.docx]

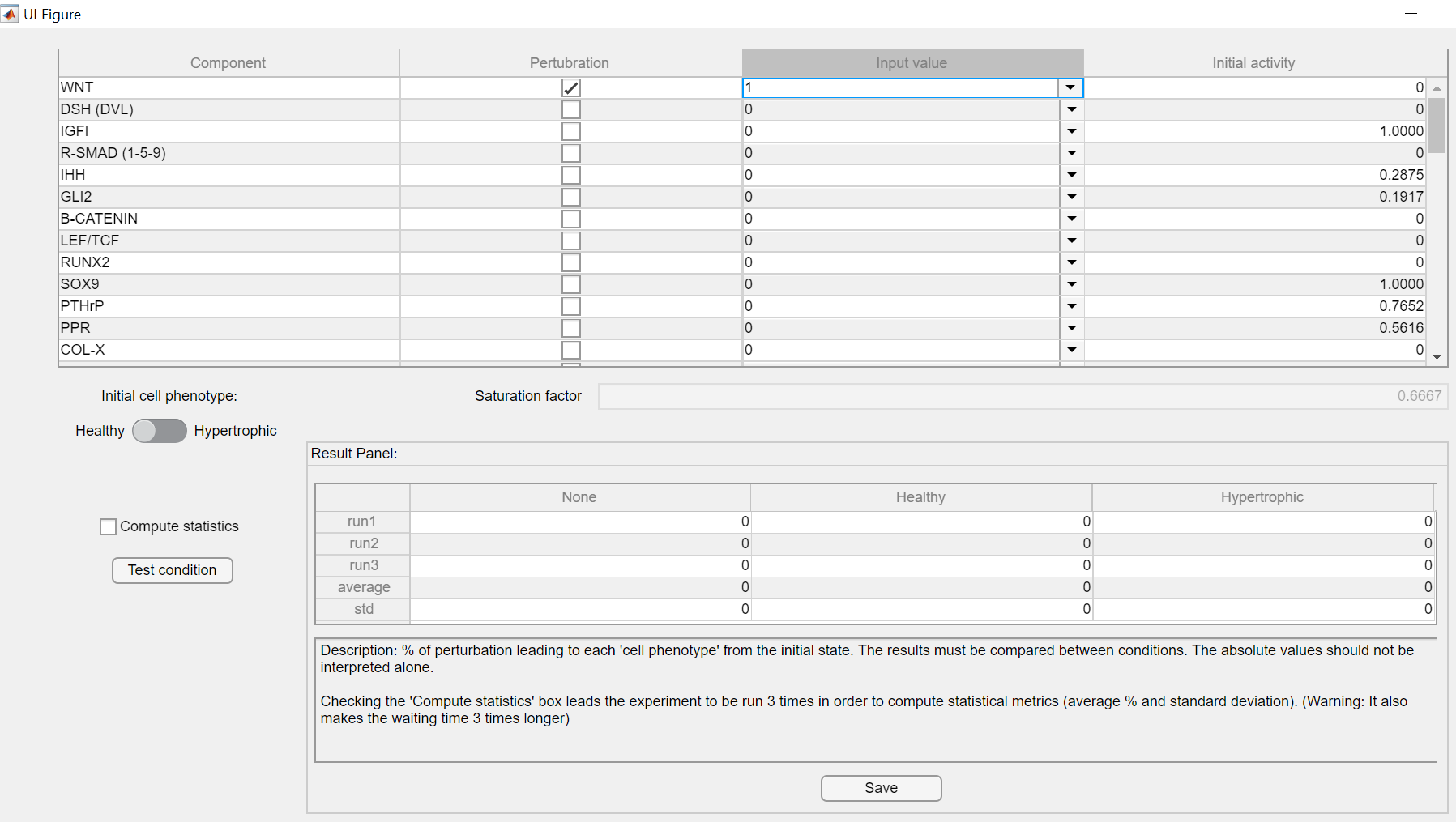


Fig. S3. Screenshot of the user-friendly interface for the virtual chondrocytes App

The standalone Matlab-based applications can be launched and used without Matlab license, provided that the compiler Matlab Runtime is installed (<https://nl.mathworks.com/products/compiler/matlab-runtime.html>). The virtual chondrocyte initial state can be set as healthy or hypertrophic, allowing the user to test any scenarios. All the 60 components may be perturbed alone or in any sort of combination by forcing the variables to take a value in the interval [0:1], with a step of 0.1. The most left column indicate the value of the variable in the selected initial state, for information. Obviously, applying a perturbation that is equal to the initial value of the variable will not affect the system. Once the setting are done, the user can apply the experimental condition by pushing the button ‘Test condition’ and the percentage of transition towards each of the possible basal stable states (i.e. ‘None’, ‘Healthy’ and ‘Hypertrophic’) is computed. If the ‘Compute statistics’ box is ticked, then the experiment is repeated 3 times and the average and standard deviations are displayed (variation occurs due to the stochastic nature of the model). The results may be exported and saved in an excel file via the ‘Save’ button. The application can be installed with the executable file on the GitHub repository, [<https://github.com/Rapha-L/Insilico_chondro.git>]. No Matlab license is required, however, the operating system should be able to support the Matlab software. For Linux users, a Windows virtual machine may be used.
