# Supplementary material for: An integrated in silico-in vitro approach for identifying therapeutic targets against osteoarthritis
Source: BMC Biol. 2022 Nov 9;20:253. doi: 10.1186/s12915-022-01451-8 (PMC9648005; doi:10.1186/s12915-022-01451-8)
Supplement: Supplementary file 12 — Additional file 12: Table S4. Targets and associated small molecules or growth factors for in vitro validation. The first row indicates the targets to be perturbed, [+] stands for activation while [-] stands for inhibition. The name (resp. cat number) of the small molecule or growth factor employed to achieve that effect is indicated in the row called ‘Molecule name’ (resp. ‘Cat n°’). [file 12915_2022_1451_MOESM12_ESM.docx]

**Table S4:** **Targets and associated small molecules or growth factors for *in vitro* validation**

| Intended target | Effect | Molecule Name | Cat n° | Short name |
| --- | --- | --- | --- | --- |
| **PKA** | + | Forskoline | Axon 2264 | Fork |
| **SMAD3** | + | Activin | 338-AC-010 | Activin |
| **FGFR1** | - | PD161570 | Axon 2098 | PD16 |
| **WNT** | - | IWP2 | 72122 | IWP2 |
| **ERK1/2** | - | PD0325901 | Axon 1408 | PD03 |
| **IGFI/IGFIR** | + | IGFI | 791-MG-050 | IGFI |
| **ALK5** | + | TGF-β1 | 100-21-50 | TGF |
| **BMP** | - | LDN-193189 | Axon 1509 | LDN |
| **HDAC4** | + | ITSA1 | 18681 | ITSA1 |

The first row indicates the targets to be perturbed, [+] stands for activation while [-] stands for inhibition. The name (resp. cat number) of the small molecule or growth factor employed to achieve that effect is indicated in the row called ‘Molecule name’ (resp. ‘Cat n°’)
