# Supplementary material for: An integrated in silico-in vitro approach for identifying therapeutic targets against osteoarthritis
Source: BMC Biol. 2022 Nov 9;20:253. doi: 10.1186/s12915-022-01451-8 (PMC9648005; doi:10.1186/s12915-022-01451-8)
Supplement: Supplementary file 13 — Additional file 13: Fig. S4. Decision tree summarizing the variable updating scheme employed in algorithm to simulate the in silico chondrocyte. Each biological component is represented by the gene expression level (slow variable) and the protein activity potential (fast variable). Variables are updated based on the rules stored in the model’s equations. First, fast variables are updated in random order, when a pseudo-stable state is reached and that all fast variables have been updated, the next random chosen slow variable is updated. This goes on until a state that is stable both at the fast and slow level is reached. This is the final stable state. A state is considered stable if further variable updates do not bring further changes for any of the variables, with a predefined tolerance interval. The order in which variables are updated is random, thereby generating some stochasticity in the model. Within the fast (resp. slow) updating loops, variables are updated asynchronously (meaning the one after the others) according to the rules defined in the system of equations and in a random order. For some systems (i.e. set of equations) cyclic attractors may arise, meaning that the system never reaches a fixed stable state but oscillates between several states. This situation did not occur in the current study. [file 12915_2022_1451_MOESM13_ESM.docx]

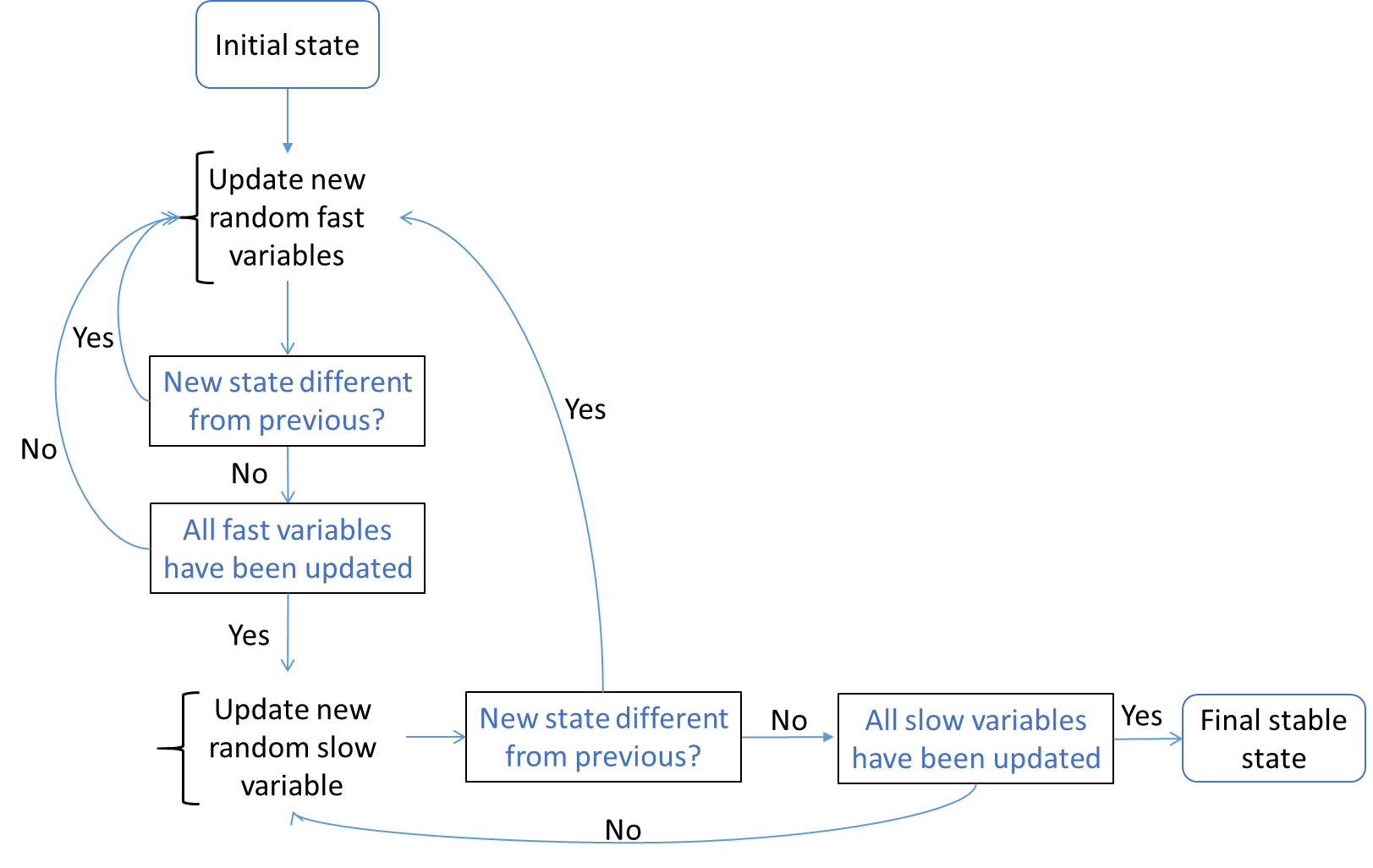


Fig. S4. Decision graph summarizing the variable updating scheme employed in algorithm to simulate the *in silico* chondrocyte

Each biological component is represented by the gene expression level (slow variable) and the protein activity potential (fast variable). Variables are updated based on the rules stored in the model’s equations. First, fast variables are updated in random order, when a pseudo-stable state is reached and that all fast variables have been updated, the next random chosen slow variable is updated. This goes on until a state that is stable both at the fast and slow level is reached. This is the final stable state. A state is considered stable if further variable updates do not bring further changes for any of the variables, with a predefined tolerance interval. The order in which variables are updated is random, thereby generating some stochasticity in the model. Within the fast (resp. slow) updating loops, variables are updated asynchronously (meaning the one after the others) according to the rules defined in the system of equations (see **Fig S5** and Supplementary text in **Additional file 2**) and in a random order. For some systems (i.e. set of equations) cyclic attractors may arise, meaning that the system never reaches a fixed stable state but oscillates between several states. This situation did not occur in the current study, but in case it does for a new set of equation, this could be handled in different ways depending on the purpose of the study. For instance, the difficulty of finding these cyclic attractors can be circumvented by adapting the algorithm and looking at the qualitative nature of the attractors (rather than the particular change of activities of each variables). When a simulation does not converge to a singleton attractor after, say 500 updates (of slow variables) the activities of the SOX9 and RUNX2 variables can be averaged over the last 200 states. The number of updates should be chosen to be significantly higher than the time typically needed to converge for singleton attractors, to prevent these attractors from being misclassified. With this information, the attractors could be classified qualitatively (e.g. as RUNX2 positive, SOX9 positive, a combination of both, or neither) see [1] for further information.

Reference:

1. Kerkhofs J. Chondrogenic Differentiation in the growth Plate: a Computational Modelling Approach. [Doctoral thesis] KU Leuven, University of Liege. 2015.
