# Supplementary material for: An integrated in silico-in vitro approach for identifying therapeutic targets against osteoarthritis
Source: BMC Biol. 2022 Nov 9;20:253. doi: 10.1186/s12915-022-01451-8 (PMC9648005; doi:10.1186/s12915-022-01451-8)
Supplement: Supplementary file 14 — Additional file 14: Fig. S5. Illustration of the algorithm for the asynchronous updating of variables with a simplified (3 nodes) example network. The network represents interactions happening in one of the subnetworks (protein= fast reactions or genetic = slow reactions). Inhibitions are represented in red and activations are in black. The mathematical rules corresponding to the network are displayed. If the rules result in a value lower than 0 (resp. higher than 1), the value is brought back to 0 (resp. 1). For this example, 3 different initial states are inputted and each of the three variables is updated asynchronously. The order in which variables are updated is random. The system reaches a stable state when the next update gives the same state as in the previous time step and that all variables were screened in the random ordering list. That state is a pseudo-stable state if the rules were describing fast reactions, in that case, a new slow variable can be updated (see Fig. S4.). However it is a final stable state if the rules were describing slow interactions since it would mean that the system had first reached a pseudo-stable state at the fast level and would now be stable at the slow level too. The example illustrates that different initial states may reach the same final state but also that the same initial state (e.g. [1 0 1] ) can reach different final states, depending on the order in which variables are updated, thereby introducing stochasticity in the system. [file 12915_2022_1451_MOESM14_ESM.docx]

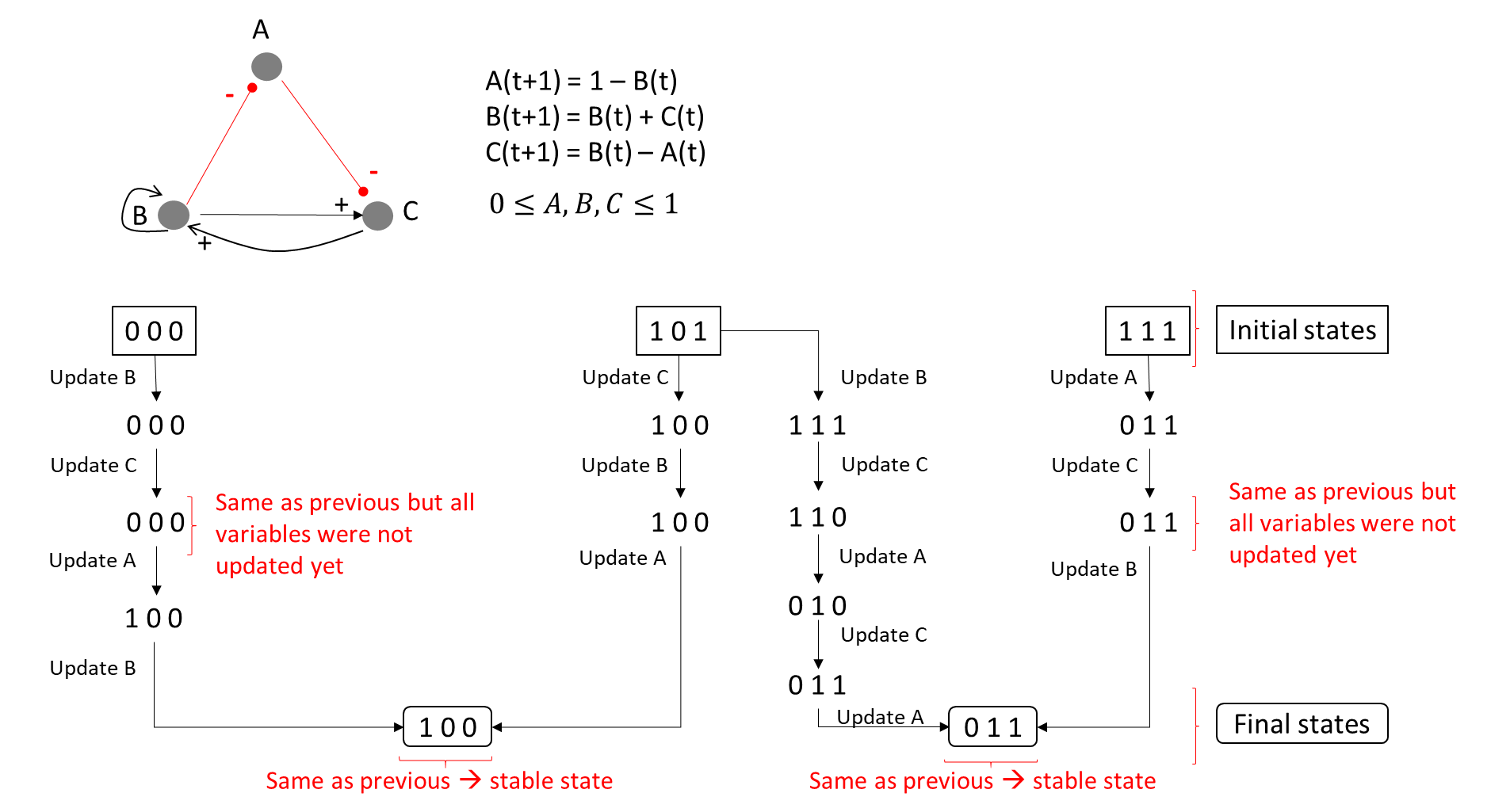


Fig. S5. Illustration of the algorithm for the asynchronous updating of variables with a simplified example network

The network represents interactions happening in one of the subnetworks (protein= fast reactions or genetic = slow reactions). Inhibitions are represented in red and activations are in black. The mathematical rules corresponding to the network are displayed. If the rules result in a value lower than 0 (resp. higher than 1), the value is brought back to 0 (resp. 1). For this example, 3 different initial states are inputted and each of the three variables is updated asynchronously. The order in which variables are updated is random. The system reaches a stable state when the next update gives the same state as in the previous time step and that all variables were screened in the random ordering list. That state is a pseudo-stable state if the rules were describing fast reactions, in that case, a new slow variable can be updated (see **Fig. S4**.). However it is a final stable state if the rules were describing slow interactions since it would mean that the system had first reached a pseudo-stable state at the fast level and would now be stable at the slow level too. The example illustrates that different initial states may reach the same final state but also that the same initial state (e.g. [1 0 1] ) can reach different final states, depending on the order in which variables are updated, thereby introducing stochasticity in the system.
