# Supplementary material for: An integrated in silico-in vitro approach for identifying therapeutic targets against osteoarthritis
Source: BMC Biol. 2022 Nov 9;20:253. doi: 10.1186/s12915-022-01451-8 (PMC9648005; doi:10.1186/s12915-022-01451-8)
Supplement: Supplementary file 15 — Additional file 15: Fig. S6. Sensitivity analysis: impact of the number of initialization on the canalization results during the Monte Carlo. The percentage of random initialization reaching each attractor is displayed with the Sox9 positive state (healthy) in orange, the Runx2 positive state in blue and the None in grey. Data labels indicate the absolute amount of state reaching the attractors. None of initializations reached an alternative attractor, even for higher amount of random initializations The number of initialization has no significant impact on the basal canalization and 10.000 initializations were considered sufficient to screen the state space in the current study. [file 12915_2022_1451_MOESM15_ESM.docx]

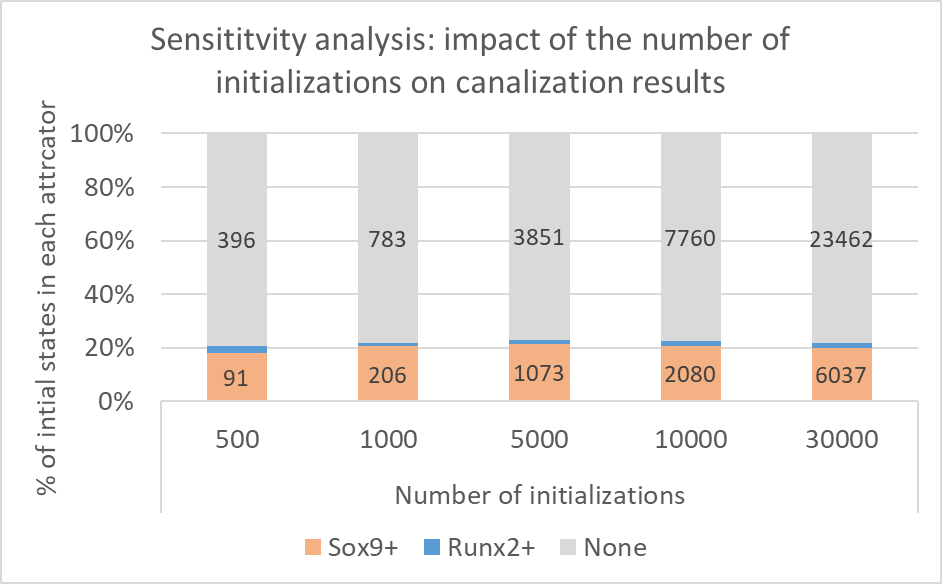


Fig. S6. Sensitivity analysis: impact of the number of initializations on the canalization results during the Monte Carlo analysis. The percentage of random initializations reaching each attractor is displayed with the Sox9 positive state (healthy) in orange, the Runx2 positive state in blue and the None in grey. Data labels indicate the absolute amount of state reaching the attractors. None of initializations reached an alternative attractor, even for higher number of random initializations The number of initializations has no significant impact on the basal canalization and 10.000 initializations were considered sufficient to screen the state space in the current study.
