# Supplementary material for: An integrated in silico-in vitro approach for identifying therapeutic targets against osteoarthritis
Source: BMC Biol. 2022 Nov 9;20:253. doi: 10.1186/s12915-022-01451-8 (PMC9648005; doi:10.1186/s12915-022-01451-8)
Supplement: Supplementary file 16 — Additional file 16: Fig. S7. Pseudo-time evolution of variables during simulations. The sequence of variable updating over each time steps after introducing a perturbation from the healthy state was saved as a timeseries and plotted. It shows the discrete behavior of the simulation and that a steady state is reached way before the duration of the perturbation (1000 time steps) is reached in such a way that maintain the perturbation longer would not change the output of the simulation once the perturbation is released. [file 12915_2022_1451_MOESM16_ESM.docx]

**
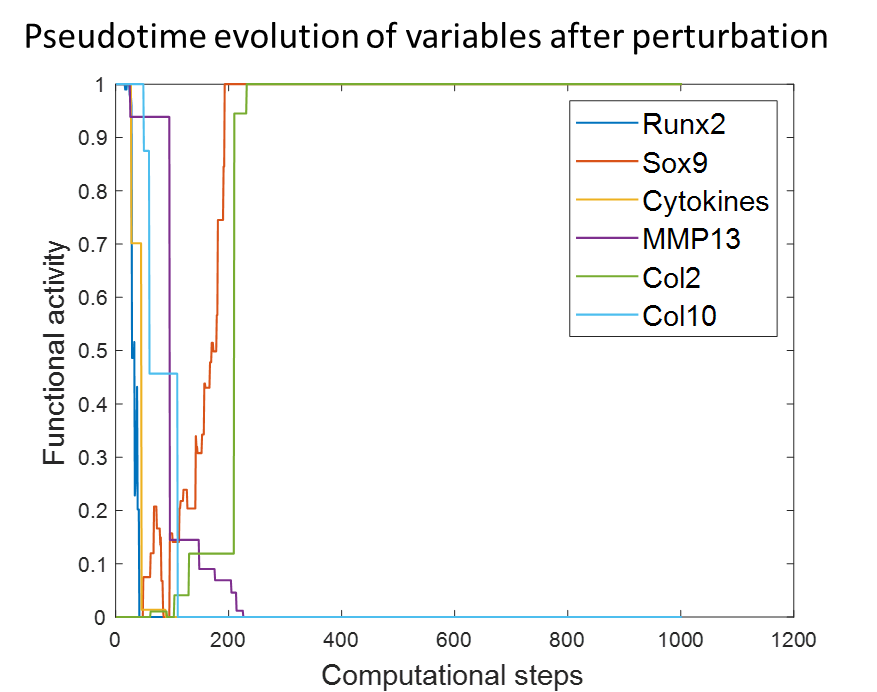
**

**Fig. S7. Pseudotime evolution of variables during simulation after PKA activation with FGFR1 inhibition.**

The sequence of variable updates at each time steps after introducing a perturbation from the hypertrophic state was saved as a timeseries and plotted. The initial state is a hypertrophic chondrocyte and the perturbation (PKA activation and FGFR1 inhibition) is applied a t=0. The pseudo-time evolution is displayed for SOX9, RUNX2, MMP13, Pro-inflammatory cytokines, Collagen II and X. It shows the discrete behavior of the simulation and highlight that a steady state is reached way before the duration of the perturbation is reached, in such a way that maintaining the perturbation longer would not change the output of the simulation.
